# Cancer Nanovaccines: Mechanisms, Design Principles, and Clinical Translation

**DOI:** 10.1021/acsnano.4c15765

**Published:** 2025-04-09

**Authors:** Gabriel de Camargo Zaccariotto, Maria Julia Bistaffa, Angelica Maria Mazuera Zapata, Camila Rodero, Fernanda Coelho, João Victor Brandão Quitiba, Lorena Lima, Raquel Sterman, Valéria Maria de Oliveira Cardoso, Valtencir Zucolotto

**Affiliations:** † Nanomedicine and Nanotoxicology Group, São Carlos Institute of Physics, University of São Paulo, São Paulo 13566-590, Brazil

**Keywords:** cancer nanovaccines, nanoparticle-based immunotherapy, nanotechnology, immune activation, antitumor immune response, nanoparticle design, immunotherapy, nanooncology, clinical translation

## Abstract

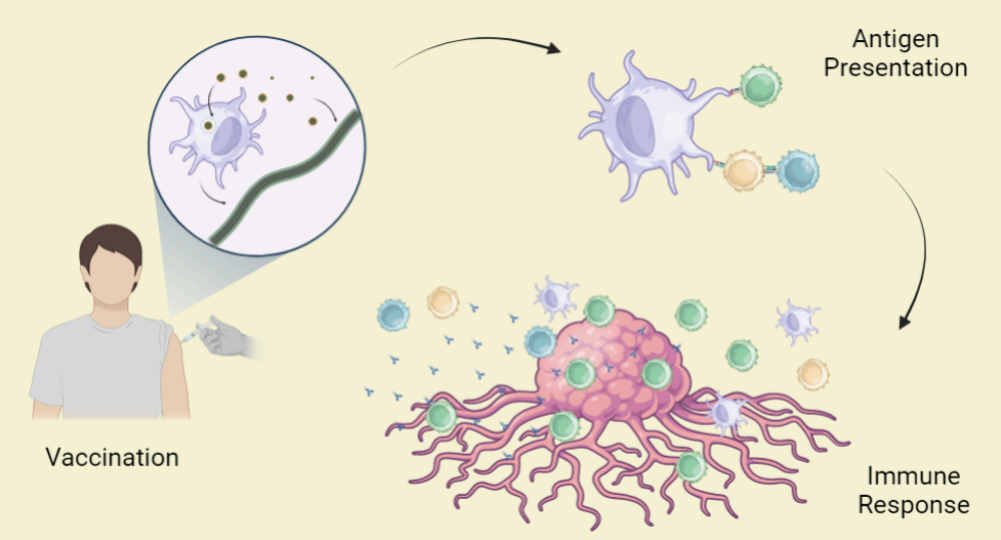

Cancer immunotherapy has transformed the landscape of oncological treatment by employing various strategies to teach the immune system to eliminate tumors. Among these, cancer nanovaccines are an emerging strategy that utilizes nanotechnology to enhance immune activation in response to tumor antigens. This review addresses the principles behind the different technologies in this field aimed at generating a robust and effective immune response. The diversity of strategies adopted for the design of nanovaccines is discussed, including the types of active agents, nanocarriers, their functionalizations, and the incorporation of adjuvants. Furthermore, strategies to optimize nanoparticle formulations to enhance the antigen presentation, target immune cells, and organs and promote strong and durable antitumor responses are explored. Finally, we analyze the current state of clinical application, highlighting ongoing clinical trials and the future potential of cancer nanovaccines. The insights presented in this review aim to guide future research and development efforts in the field, contributing to the advancement of more effective and targeted nanovaccines in the fight against cancer.

## 1 Introduction

### 1.1 Cancer Vaccines

Vaccination has long been a cornerstone of public health, achieving remarkable successes such as the eradication of smallpox—whose vaccine was introduced in 1796—and the control of polio and diphtheria.^1^ These historical achievements have saved countless lives and redefined global health. The convergence of advancements in immunology, molecular biology, and nanotechnology has further revolutionized the field of vaccines. This progress now holds the potential to radically transform the treatment of cancer, one of the most complex and challenging diseases, marking the advent of a new era in oncological therapy.^2^ Despite advances in diagnosis and treatment, cancer still represents a significant challenge due to its ability to evade the immune defense system and its resistance to conventional treatments.^3^ Traditionally, cancer therapy is based on surgical resection followed by chemotherapy and radiotherapy, but these treatments can be highly debilitating and often only offer modest improvements in patient survival due to their limitations in terms of specificity and efficacy. As a result, immunotherapies, such as cancer vaccines, have emerged as a new paradigm by harnessing the immune system’s ability to attack cancer cells.

Cancer vaccines utilize a variety of antigens that are overexpressed on tumor cells compared to normal cells or that are derived from specific somatic mutations in the tumor. These antigens can be derived from peptides, recombinant proteins, DNA, and mRNA, all of which have the potential to induce a targeted immune response.^4,5^ These vaccines are classified into preventive and therapeutic types. Preventive vaccines aim to stop tumor growth and proliferation before they manifest clinically, while therapeutic vaccines are designed to treat existing tumors by sensitizing the immune system to recognize and destroy cancer cells.^5^ Cancer nanovaccines are primarily administered through intramuscular (IM), subcutaneous (SC), and intravenous (IV) routes. The choice of administration route directly influences the biodistribution of the immunogen and the activation of the immune response. After administration via IM or SC, nanoparticles are rapidly taken up by antigen-presenting cells (APCs) promoting their internalization and processing. These nanoparticles can also migrate through the lymphatic system and bloodstream, reaching secondary lymphoid organs, such as lymph nodes and the spleen, where T and B cell activation occurs, an essential step for inducing a robust and sustained immune response.^6^ In IV administration, nanoparticles in the bloodstream can be captured by circulating APCs or by resident APCs in the spleen and lymph nodes throughout the body, initiating the vaccine response.^7^ However, they are also subject to clearance by the mononuclear phagocyte system (MPS) and accumulation in the liver.^8,9^ Beyond traditional parenteral routes, *in situ* (intratumoral) administration has been widely explored for cancer nanovaccines. This strategy stimulates an immune response within the tumor microenvironment (TME) by using intratumoral injections of immunomodulators to take advantage of the abundance of tumor-associated antigens present in the tumor, enhancing immune responses while minimizing systemic adverse effects. Studies have shown that intratumoral application can improve antigen uptake by APCs, stimulate inflammation at the tumor site, and increase T-cell activation against cancer cells.^10−12^

The first cancer vaccine emerged in 1893 when Dr. William B. Coley introduced Coley’s toxins (derived from inactivated bacteria), administered directly into sarcoma tumors.^13^ Since then, significant advances have been made with vaccines targeting a wide range of cancer types and the advances in cancer vaccine technology are evident in approval vaccines and numerous clinical trials.

### 1.2 Drawbacks in Cancer Vaccines

Cancer vaccines face several challenges, primarily limited efficacy in clinical trials,^14^ due to difficulties in designing optimal delivery platforms, selecting effective combination therapies, overcoming immune suppression within the TME, and eliciting a robust T-cell immune response.^13^

To improve cancer vaccines efficiency, selecting appropriate tumor antigens is critical; such antigens must be sufficiently immunogenic to trigger a robust immune response without causing adverse reactions. Additionally, immunosuppression within the TME presents a significant obstacle, as tumors can create conditions that effectively inhibit the activity of T cells and other immune cells.^3^ This is particularly relevant in distinguishing between “hot tumors”, which are inflamed and infiltrated with immune cells, and “cold tumors”, which lack significant immune cell infiltration and are more challenging to treat.^15^ Moreover, there are technical challenges also related to the effective delivery of antigens. Vaccines must reach APCs efficiently to be processed and presented to the immune system. This requires delivery systems that protect the antigens while ensuring their effective release at the desired site. Variability in immune responses among individuals also poses a challenge, as some patients respond well to vaccination while others show little or no response.^3^

The design process may require customization for each patient, based on detailed genetic sequencing, which can be both time-consuming and expensive, being another critical point. This raises costs and complicates the logistics of manufacturing and large-scale distribution. Additionally, there are considerable clinical and regulatory challenges. Extensive clinical trials are necessary to ensure that the vaccines are safe and effective, which requires time, resources, and rigorous coordination to meet regulatory standards and obtain approval for clinical use.^16^ Despite these challenges, vaccines combined with immune checkpoint inhibitors—drugs that block immune system proteins such as programmed cell death protein 1 (PD-1) and programmed death-ligand 1 (PD-L1)—have shown promising responses in reported cases.^7^ These inhibitors allow T cells to recognize and attack tumor cells, significantly increasing the effectiveness of vaccines.^15,17^ However, several challenges remain on clinical efficacy of cancer vaccines.

Nanotechnology stands out in this scenario by offering strategies to overcome the above-mentioned limitations through the use of nanocarriers (NCs). Designed to improve the delivery and presentation of tumor antigens, the NCs increase the precision and effectiveness of immune activation, as well as specifically targeting the lymph nodes, where a large proportion of immune cells can be efficiently activated. In addition, modifying the surface of NCs allows for the codelivery of adjuvant molecules that can stimulate a robust immune response, directly addressing the problem of immunosuppression caused by the tumor.^4,17,18^ Therefore, nanotechnology not only promises to enhance the efficacy of existing vaccines but also opens new opportunities for the development of more efficient vaccination strategies that are less susceptible to tumor resistance mechanisms. This innovative technology has the potential to transform the field of cancer immunotherapy, offering more effective and personalized treatments.

In this review, the emerging field of nanovaccines in cancer immunotherapy will be explored, highlighting innovations and advances that are shaping the present and future of oncological treatment. Initially, the concept of nanovaccines and their differences compared to conventional vaccines is introduced. Next, the aspects related to the different types of nanovaccines based on their mechanisms for stimulating the immune response, as well as the types of NCs and their modifications aimed at robust and long-lasting immune activation will be discussed. The latest data on nanovaccines in clinical trials I–III will be presented. This review provides a comprehensive overview of how nanovaccines are emerging as one of the most promising frontiers in the fight against cancer, promising to transform the landscape of oncological therapy shortly.

## 2 Nanovaccines

Represented by an advancement from conventional vaccines, nanovaccines apply the power of nanotechnology to redefine immunization strategies. It relies on engineered NCs as vehicles for antigens, adjuvants, or other immunomodulatory agents.^19−21^ These NCs can be precisely tailored in terms of size, shape, surface chemistry, and composition to optimize antigen delivery, immunogenicity, and targeting specificity. Typically, nanovaccines are composed of three fundamental elements: antigens, adjuvants, and NCs.^22^ Each component plays a crucial role in shaping the immune response, from priming the immune system to recognizing tumor-specific targets to amplifying and sustaining antitumor immunity, working synergistically to enhance the immune response against cancer cells.^19^

Antigens, one of the key components of vaccines, stimulate the immune system to recognize and elicit a response against specific targets. In the context of cancer vaccines, antigens can originate from natural tumor antigens, mRNA, synthetic peptides, and DNA encoding tumor antigens.^23^ These antigens are commonly divided into two main classes: tumor-associated antigens (TAAs) and tumor-specific antigens (TSAs).^24^ TAAs are proteins found on the surface of both cancerous and noncancerous cells. While not unique to cancer, they are often overexpressed on tumor cells, making them a target for immune attack.^25^ Examples of TAAs include the following: MART-1, found in melanocytes and melanomas; HER2/neu, overexpressed in some types of breast cancer; AFP (α-fetoprotein) in liver tumors; CEA (carcinoembryonic antigen) in colorectal cancer.^26−28^ However, TAAs can sometimes lead to immune tolerance, where the body fails to recognize them as a threat, or trigger a response against healthy cells. TSAs, on the other hand, arise from genetic mutations within cancer cells. These mutations are unique to each tumor and nanovaccines containing TSAs offer a more precise strategy, targeting the specific vulnerabilities of each cancer.^29^ Examples of exclusive antigens are those resulting from mutations in the TP53, KRAS, and BRAF genes.^30−32^ However, some challenges still persist, such as the high variability between malignancies and patients, making it difficult to develop a one-size-fits-all vaccine, requiring the constant use of techniques like genetic sequencing and bioinformatics tools for each individual patient. This requirement increases the complexity of the process and associated costs.^33,34^

Adjuvants are the second essential component of nanovaccines. Whether molecular or nanoparticle-based, adjuvants enhance the immune response to antigens by activating APCs and promoting inflammation at the target site.^23^ In nanovaccines, adjuvants can be incorporated either as molecular adjuvants, such as toll-like receptor (TLR) agonists or cytokines, or as NCs-based adjuvants, such as liposomes or polymeric.^22^ These adjuvants play a crucial role in amplifying the immune response, improving antigen presentation, and promoting the maturation of immune cells, ultimately leading to enhanced vaccine efficacy.

**Figure 1:**
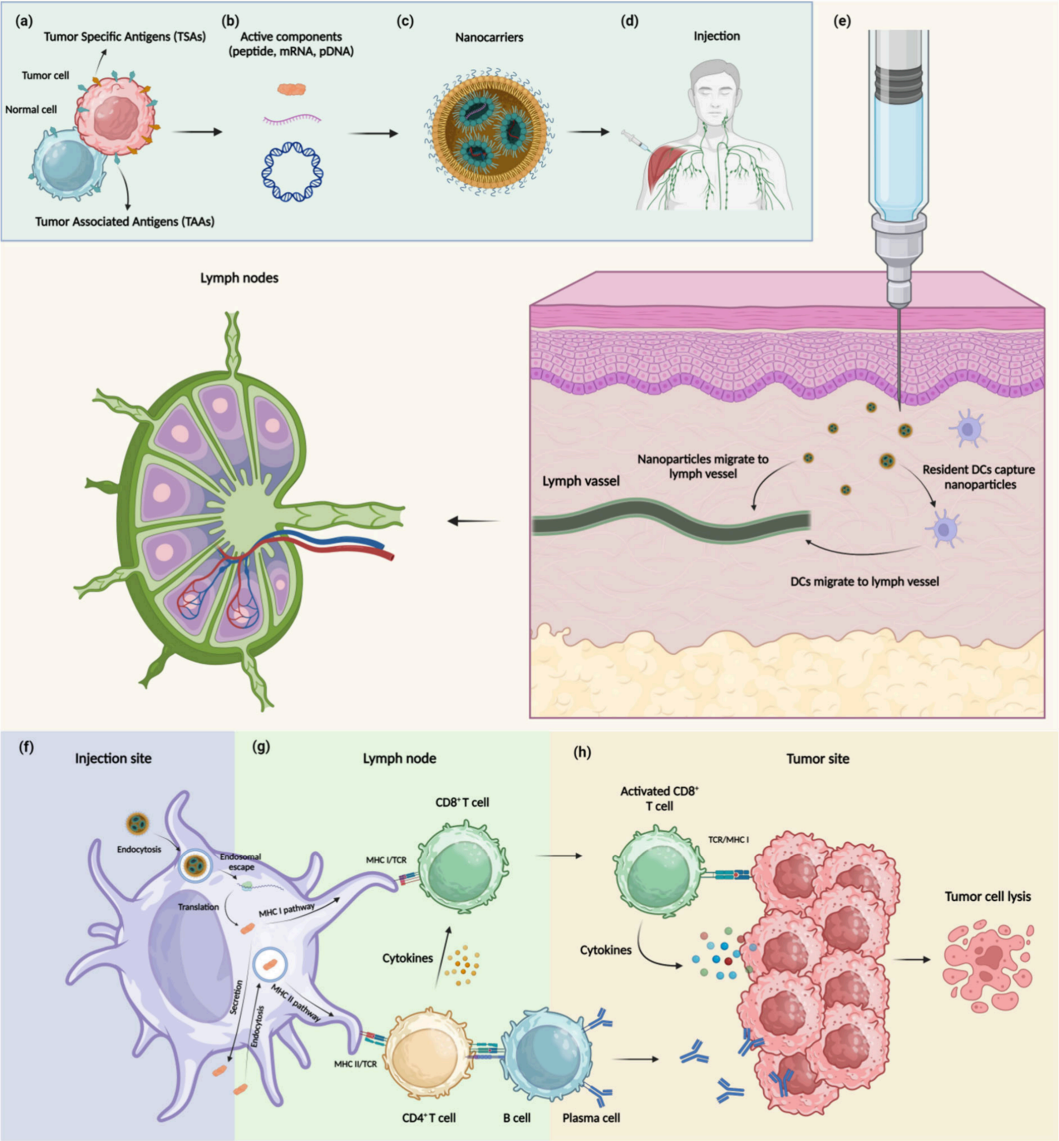
General scheme how nanovaccines work. (a) Vaccine antigens can be TAAs (common to both normal and tumor cells and overexpressed in tumor cells) or TSAs (exclusive to tumor cells); (b) antigens can be delivered as different active agents, such as peptides, mRNA, or pDNA; (c) these active agents are encapsulated in NCs; (d) administration (e.g., intramuscularly); (e) upon administration, smaller nanoparticles tend to migrate directly to the lymphatic vessels, while larger nanoparticles tend to be captured by resident DCs, which then migrate to the lymphatic system; (f) at the injection site, nanoparticles undergo endocytosis by DCs; (g) after processing the active agents, DCs present the antigen(s) via MHC class I and II pathways to CD8+ T and CD4+ T lymphocytes, respectively; (h) activated lymphocytes and specific antibodies recognize the antigen(s) on tumor cells. Created by BioRender.com.

The third crucial component of nanovaccines are the NCs, delivery vehicles that encapsulate antigens and/or adjuvants, protecting them from degradation, facilitating their controlled release, and promoting their targeted delivery to specific cells or tissues.^35^ They can be composed of lipids, polymers, or inorganic materials, each offering unique advantages in terms of stability, biocompatibility, and customization.^29^ These NCs can be engineered to present desirable properties such as surface modifications for targeting specific receptors on immune cells or stimuli-responsive release mechanisms triggered by the TME. By serving as versatile platforms for vaccine delivery, NCs enable precise control over antigen/adjuvant release kinetics and enhance the therapeutic efficacy of nanovaccines against cancer. Figure 1 depicts a scheme of how nanovaccines work.

## 3 Types of Nanovaccines

### 3.1 Peptide Vaccines

Peptide vaccines are important in immunotherapy, using synthetic peptides as antigens to stimulate the immune system against pathogens and tumor cells. They are composed of short amino acid sequences that mimic epitopes, providing specificity, safety and an easy production. Synthetic peptide production is relatively straightforward and cost-effective, making its processing quicker and scalable. Additionally, it is possible to customize for different cancer mutations, allowing for personalized cancer vaccines. Importantly, as synthetic peptides are not live pathogens, they do not pose any risk of disease and are suitable for immunocompromised individuals.^36^

After administration, peptide vaccines are processed by APCs, such as dendritic cells (DCs). APCs present peptides on major histocompatibility complex (MHC) molecules. Cytotoxic T lymphocytes (CTLs) recognize peptides that are displayed by MHC class I molecules, targeting and removing infected or neoplastic cells. Simultaneously, helper T cells recognize peptides presented on MHC class II molecules, which is an essential step for initiating and modulating immune response, including the activation of B cells and subsequent antibody production.^37^

Despite their promise, peptide vaccines face several limitations. Peptides themselves often lack sufficient immunogenicity, needing adjuvants and delivery systems to enhance their effectiveness. The selection of adequate epitopes is critical, requiring detailed knowledge of the pathogen’s or tumor’s antigenic structure and the host’s immune response. Additionally, peptide vaccines are subject to human leukocyte antigen (HLA) restriction, meaning their effectiveness can vary among individuals with different HLA types. Furthermore, peptides are susceptible to degradation by proteases in the body, requiring stable formulations and efficient delivery methods. Ongoing research in adjuvant development, delivery systems, and epitope mapping continues to advance peptide vaccine technology, aiming to overcome these challenges and realize their full potential in immunotherapy.^38^

### 3.2 Nucleic Acid Vaccines

Nucleic acid vaccines use DNA or RNA genetic sequences to instruct host cells to produce a specific antigen, triggering an immune response. These types of vaccines have gained prominence due to their ability to induce immunity efficiently and quickly.^39,40^ DNA vaccines contain plasmids (pDNA) that encode the antigen of interest. When the vaccine containing the sequence is administered, the plasmids must enter the cell and nucleus so that the pDNA can be transcribed into mRNA, which will be translated into proteins that enable antigen presentation.^41,42^ In the case of RNA vaccines, the main component is synthetic mRNA. Once administered, the mRNA must cross the cell membrane, escape the endosome, and be directly translated into antigenic proteins. These vaccines advance one step further in antigen production, starting the process at the translation step, without needing to cross the cell nucleus, as the molecule will be processed by ribosomes in the cytoplasm. This represents an important advantage over pDNA vaccines, as it allows transient expression of the antigen and eliminates the chances of integration or mutational insertion into the host’s genetic material, ensuring safety.^43,44^ In addition, the COVID-19 pandemic greatly accelerated the development and release of multiple RNA vaccines, showcasing the platform’s remarkable adaptability, safety, and potential immunogenicity, making them appealing and effective immunotherapeutic platforms against cancer.^40^

### 3.3 Molecular Adjuvants

Optimizing vaccine efficacy through modulation of the immune response to the vaccine antigen is one of the primary functions of molecular adjuvants. These adjuvants not only increase the antigen’s immunogenicity but can also direct the immune response toward a desired profile, such as Th1, Th2, or cytotoxic T cell responses. This is essential for optimizing vaccine efficacy, especially in cases where the antigen alone is insufficient to generate a robust and long-lasting immune response.^45,46^ Furthermore, enhancing cellular uptake, endosomal escape, and cross-presentation of antigens are critical actions mediated by molecular adjuvants, leading to more robust immune responses. Studies have shown that adjuvants such as fluorinated diphenylalanine peptides can increase self-assembly capability, antigen-binding affinity, and DCs maturation, resulting in strong cellular immunity and long-term immune memory against tumors.^47^ Additionally, lipid nanovaccines have been developed to codistribute immunomodulators and peptides effectively, inducing potent prophylactic effects *in vivo* and tumor suppression through enhanced expansion and activation of antigen-specific CD8+ T cells.^48^ Moreover, self-adjuvanting polymer NCs have demonstrated significant tumor growth inhibition and prolonged survival rates, promoting DC maturation, cytotoxic T cell expansion, and causing abscopal effects without the need for additional adjuvants.^49^

Among the different types, CpG oligodeoxynucleotides (ODNs) are DNA sequences containing unmethylated cytosine–phosphate–guanine (CpG) dinucleotides. Recognized by Toll-like receptor 9 (TLR9), primarily expressed in DCs and B cells, the activation of TLR9 by CpG ODNs results in a robust immune response, including the production of pro-inflammatory cytokines and the activation of T and B cells.^50,51^ TLR agonists are pattern recognition receptors (PRRs) that detect pathogens and activate innate immune responses. Besides TLR9, other TLRs, such as TLR3, TLR4, and TLR7/8, have been targeted for adjuvant development, such as lipopolysaccharides (LPSs) for TLR4, polyinosinic acid (poly(I)) for TLR3, and imidazoquinolines for TLR7/8.^52^ Similarly, polyethylenimines (PEIs), cationic polymers capable of forming complexes with nucleic acids and facilitating cellular delivery, have been shown to function as TLR agonists. PEIs promote DC maturation, T cell activation, and enhance nanovaccine-induced immune responses by inducing cytokine production and balancing Th1/Th2 responses.^53^

Another important class of adjuvants are stimulator of interferon gene (STING) agonists, crucial components of innate immunity, which target the STING to mediate the detection of cytosolic DNA. These agonists, such as cyclic dinucleotides (CDNs), activate the STING pathway, leading to the production of type I interferons and other cytokines, thereby promoting a strong antiviral and antitumor immune response.^54,55^

Finally, cytokines are signaling proteins that modulate the immune response. The inclusion of cytokines as adjuvants in nanovaccines can direct the immune response toward a desired profile. For instance, interleukin-12 (IL-12) can promote Th1 responses, while -14 (IL-4) can favor Th2 responses. Combining cytokines with NCs can significantly enhance vaccine efficacy by increasing the activation and expansion of antigen-specific T cells.^56,57^

In summary, molecular adjuvants, such as PEIs, TLR agonists, STING agonists, and cytokines, play a key role in enhancing nanovaccines by boosting targeted immune responses. These adjuvants improve the efficacy of nanovaccines, advancing immunization strategies and opening new possibilities in nanomedicine.

## 4 Nanocarriers

NCs are revolutionizing the field of nanovaccines with their unique properties. Their high surface area allows them to carry a significant amount of antigen compared to their size. Novel generations of NCs emerged to act as a shield by avoiding the rapid clearance and degradation of bioactive constituents *in vivo*, extending their circulation half-life. This enables efficient targeted delivery to specific organs, tissues, or cells, and facilitates controlled release and intracellular delivery of antigens and/or adjuvants.^22,23^

A variety of NCs have been used for cancer immunomodulation, including polymeric nanoparticles and micelles, dendrimers, solid lipid nanoparticles (SLNs), liposomes, lipid nanoparticles (LNPs), phospholipid micelles, as well as inorganic systems such as quantum dots (QDs), silica, gold, magnetic nanoparticles, and biomimetic NCs-like cell-membrane-based vesicles and virus-like particles (VLPs).^58,59^

Polymeric NCs systems have been rationally developed to inflect the role of immune populations within the TME, thus potentiating strong anticancer effects and survival outcomes. Poly(lactic-*co*-glycolic acid) (PLGA) is a polymer extensively used for performance NCs systems because it enables encapsulation of both hydrophobic and hydrophilic drugs, peptides, nucleic acids and polysaccharides.^60−62^ PLGA’s biodegradability stems from the breakdown of its ester bonds *in vivo* into metabolizable lactic and glycolic acid monomers. This inherent property, coupled with the ability to fine-tune PLGA NP size, stability, and solubility, makes them attractive for drug delivery. Specifically, polymer NPs engineered for targeted antigen delivery in immunotherapy have demonstrated the potential to induce protective immunity against cancer and infectious diseases. Although polymer NPs have shown potential, challenges such as nanoparticle aggregation, low cellular adhesion, and complex manufacturing processes— including the use of organic solvents—limit their scalability and clinical translation.^63,64^

Inorganic NCs offer multifunctionality and versatility, which are highly desirable for various delivery applications. The fluorescence of QDs, for example, allows for dynamic tracking and imaging of the nanovaccine’s action within lymph nodes, facilitated by the QDs’ ability to conjugate with antigens and adjuvants.^65^ Examples of inorganic NCs include silica NCs targeting APCs and indoximod plus oxaliplatin for pancreatic cancer.^66^ Gold glyconanoparticles have been used to create nanovaccines with gold NCs, incorporanting listeriolysin O peptide plus inhibitors anti-PD-1 or anti cytotoxic T-lymphocyte associated protein 4 (anti-CTLA-4). This combination resulted in a 97–98% regression of tumor volume in B16.F10 and B16OVA melanoma in C57BL/6 congenic mice.^67^ In addition, fluorescent magnetic nanoparticles and magnetic pull force were used to enhance antitumor efficacy. α-AP-fmNPs loaded with antigen peptide, iron oxide nanoparticles, and indocyanine green manipulate DC migrations and track their migration by multimodality imaging, with great potential applications for DC-based cancer immunotherapy.^68^ While inorganic nanoparticles offer advantages like low-toxicity, hydrophilicity, biocompatibility, and high stability compared to organic materials, their detection by immune cells often leads to phagocytosis which represents a hurdle for their FDA approval for cancer treatment, despite the promising preclinical results.^69^

Other interesting platforms include biomimetic NCs, which are cell-membrane-based vesicles derived from various cell types, including tumor cells, platelets, white or red blood cells. These vesicles retain the intricate characteristics of original cells, such as proteins and glycoproteins present in the cell membrane, making them advantageous for inducing the activation and maturation of DCs, stimulating T cells, and eliciting robust immune responses against tumors when used as carriers in nanovaccines. This strategy, which harnesses the natural biological properties of the source cell, provides homotypic targeting, immune escape properties, and longer blood circulation—advantages that cannot be replicated through synthetic means—demonstrating improved performance over bare NPs.^70,71^ Despite these advantages, challenges related to quality control, manufacturing processes, and storage stability still persist.^72^

VLPs are a strategy of self-assembled structural proteins for NCs that can offer antigen customization which lacks genetic material and is noninfectious.^73^ A prominent example is the vaccine against cervical cancer Cervarix, which was developed using a vector technology based on baculovirus expression to create antigens against HPV-16 and HPV-18 L1 VLPs. Consequently, these HPV-16 and HPV-18 L1 VLPs elicited immune responses, enhancing vaccine efficacy in cervical cancer patients.^74^ Although their production requires complex engineering, VLPs offer high biocompatibility, low toxicity, and easy, reproducible large-scale manufacturing.^73,75^

Bacteria-derived outer membrane vesicles (OMVs) are extracellular vesicles derived from the outer membrane of Gram-negative bacteria and possess a superior immunostimulatory profile, presenting various pathogen-associated molecular patterns (PAMPs) such as DNA, RNA, lipoprotein, LPS, and peptidoglycan.^76^ These PAMPs allow the activation of different subsets of DCs by stimulating various PRRs, which stands out compared to the typical use of only one type of PAMP in nanovaccines. The antigens administered via OMVs are presented by APCs and can induce an immune response against these antigens.^76,77^ Furthermore, OMVs can be fused with tumor cell membranes, allowing them to provide the target antigens.^78^ Because they are nonreplicating, OMVs have a reduced toxicity profile compared to whole bacterial vaccines. However, the loading of tumor neoantigens into OMVs still represents a major challenge, requiring the use of synthetic biology techniques, exogenous loading, or membrane fusion. Additionally, the high reactivity of some PAMPs, such as LPS, also poses a challenge, as well as the possibility of OMVs to contain immunodominant antigens that could divert the immune response.^76−78^

The literature features positive reviews that classify these NCs based on their origin or even according to the nanomaterial they are composed of.^23,29^Table 1 provides an overview of NCs, their functionalizations and the physicochemical characteristics explored to improve nanovaccine-based immunotherapy.

**Table 1:** Summary of the Nanocarriers, Their Functionalization, and Physicochemical Characteristics Explored to Improve Cancer Nanovaccines^a^

function	nanocarriers	functionalizations	physicochemical characteristics
lymph-organs targeting	• high-density lipoprotein-mimetic LNP^79^	• diacyl lipid^81^	• size: 5–100 nm (intramuscular)^85^
	• polymeric hybrid micelles^80^	• CCR7^82^	• charge: neutral or negative (intravenous)^86,87^
		• poly(ethylene glycol) (PEG)^83^	
		• Selective ORgan Targeting (SORT)^84^	
DCs targeting and/or uptake	• high-density lipoprotein-mimetic LNP^65^	• mannose^88^	• size: >100 nm (intramuscular)^85^
		• antibodies (anti-DEC205, anti-CD11c, anti-CLEC9A, anti-CD40)^89−91^	
		• thiol ligand containing both shikimoyl and guanidinyl functionalities (SGSH)^92^	
		• 12-mer Clec9a binding peptide (CBP-12)^93,94^	
		• granulocyte–macrophage colony-stimulating factor (GM-CSF)^95^	
		• MHC class II-targeting peptides^96^	
		• glycan^97^	
codelivery of molecules	• sHDL nanodisks^98^		
	• E2 nanoparticles^99, 100^		
	• LNPs^84^		
	• liposomes^101^		
	• guanidinium-containing disulfide-based thiolated nanovaccine^102^		
	• PLGA nanoparticles^60,87,103^		
	• liposomes-coated gold nanocages^104^		
	• VLPs^73^		
enhanced adaptive immune response	• PC7A nanoparticle^105^	• TLR agonists (CpG ODN, PEI, MPLA, R848 and AS04)^74,87,102,109,115,116^	
	• PLGA nanoparticle-stabilized Pickering emulsion adjuvant system (PPAS)^106^	• STING agonists (DMXAA, c-di-AMP, cGAMP, c-di-GMP, MSA-2)^117−124^	
	• asymmetric mesoporous silica nanoparticles (HTMSNs)^107^		
	• bacterial OMVs^108,109^		
	• bacterial-membrane-coated nanoparticles^110^		
	• PLGA-AC/NP and Mal-AC/NP (with radiotherapy)^111^		
	• α-alumina nanoparticles^112^		
	• VLPs^113^		
	• exosomes^114^		
endosomal escape	• LNPs^125^	• fusogenic peptides^129,129^	• charge: positive (in endosomal pH levels)^125^
	• polymeric nanomicelles^126^	• cell-penetrating peptides (CPPs)^130^	
	• DNA nanodevice/pH-activatable DNA-locking strand^127^	• pH-sensitive polymer^131^	
	• pH/enzyme-responsive nanoparticle^128^	• disulfide chemistry^132^	

a Abbreviations: AC/NP, antigen-capturing nanoparticles; AS04, MPLA and aluminum salt; CCR7, chemokine receptor type 7; c-di-AMP, bis(3′-5′)-cyclic dimeric adenosine monophosphate; c-di-GMP, bis(3′-5′)-cyclic dimeric guanosine monophosphate; cGAMP, cyclic guanosine monophosphate–adenosine monophosphate; CpG ODN, cytosine–phosphorothioate–guanine oligodeoxynucleotides; DMXAA, 5,6-dimethylxanthenone-4-acetic acid; E2, subunit of pyruvate dehydrogenase; LNP, lipid nanoparticle; Mal-AC/NP, antigen-capturing nanoparticles coated with maleimide poly(ethylene glycol); MPLA, monophosphoryl lipid A; R848, Resiquimod; MSA-2, benzothiophene oxobutanoic acid; PEI, polyethylenimine ; PLGA, poly(lactic-*co*-glycolic acid); sHDL, synthetic high-density lipoprotein; VLPs, virus-like particles.

Despite a wide number of NCs tested, clinical trials of cancer nanovaccines (Table 3) demonstrate that lipid-based NCs, primarily liposomes and LNPs, are highly prominent. Liposomes were the first delivery platform to achieve successful clinical application and are characterized by one or several closed lipid bilayers forming vesicles ranging from 20 to 1000 nm in size.^133^ They are capable of transporting hydrophilic molecules in their aqueous core, such as peptides and nucleic acids, and hydrophobic molecules in the bilayer, such as lipopeptides and adjuvants, in addition to the attachment of molecules to their surface by adsorption or chemical bonding.^101^ The main antigens transported using liposomes in clinical trials of cancer nanovaccines are peptides, such as melanoma and HPV peptides, MUC1, CEA, among others. LNPs currently represent the gold standard for the administration of nucleic acid-based vaccines.^134,135^ Neutral phospholipids, such as 1,2-distearoyl-*sn*-glycero-3-phosphocholine (DSPC), 1,2-dioleoyl-*sn*-glycero-3-phosphoethanolamine (DOPE), and sterols, such as cholesterol, are common components of liposomes and LNPs, being closely related to membrane stability. In liposomes, cationic lipids, such as dimethyldioctadecylammonium (DDA) and dioleoyl-3-trimethylammonium propane (DOTAP), are extensively used as main components.^101,135^ The composition of LNPs includes ionizable or cationic lipids, sterols, helper lipids, and PEGylated lipids.^136,137^ Among them are the ionizable lipids, such as Dlin-MC3-DMA, a component of Patisiran, and ALC-0315, component of BNT162b COVID-19 vaccine, which are crucial components, as they are protonated at low pH, becoming positively charged, but remain neutral at physiological pH.^137,138^ These characteristics allow for fewer interactions with anionic membranes when administered *in vivo*, providing biocompatibility and contributing to endosomal escape, a crucial step in the success of mRNA vaccines.^138^ Due to their importance for this class of NCs, extensive research and a number of patents are focused on developing new ionizable lipids.^102^

## 5 Advantages of Nanotechnology in Cancer Vaccines

**Figure 2:**
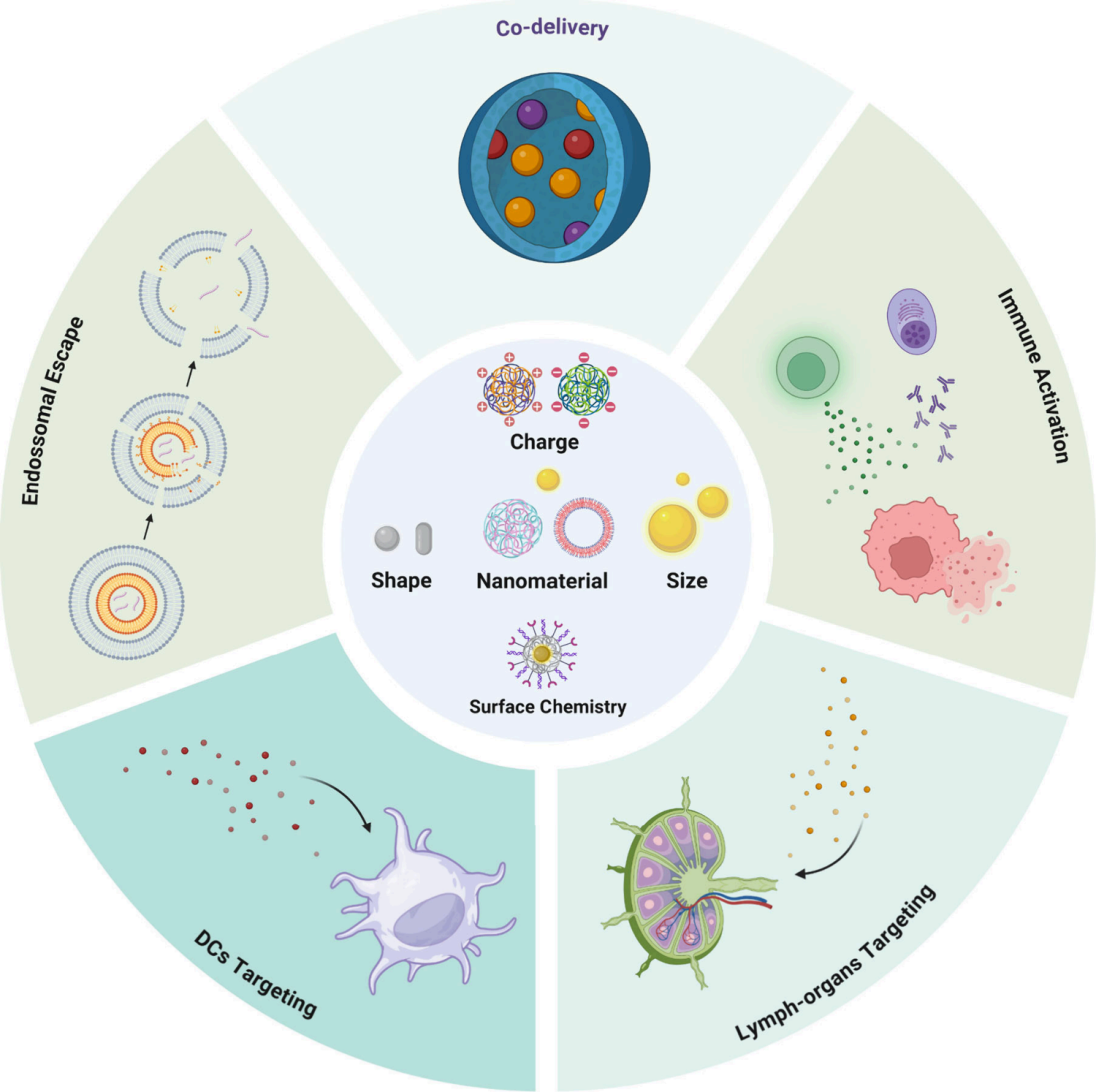
Design of nanoparticles to enhance the efficacy of cancer nanovaccines. Created by BioRender.com

The use of nanotechnology combined with cancer immunotherapy to enhance the effectiveness of cancer treatments have shown great promise. Nanovaccines offer controlled release, targeted delivery, bioavailability, increased stability, enhanced ability to stimulate robust and durable immune responses and more efficient penetration into biological barriers and tissues, facilitating the access to lymphatic nodes and TMEs.^20,139^ These advantages can be achieved by modifying the characteristics of NCs, such as size and shape, surface charge, choice of nanomaterials, and surface chemistry. Figure 2 depicts a scheme of nanoparticle design to enhance the efficacy of cancer nanovaccines.

### 5.1 Efficient Penetration in Lymphatic Tissues

The lymphatic system comprises a network of vessels and nodes that facilitate the transport of immune cells and antigens, making lymph nodes essential hubs for immune activation.^140^ Studies have shown that the size of NCs is relevant when it comes to lymphatic node delivery.^139^ Overall, particles ranging from 10 to 100 nm have demonstrated increased lymphatic uptake efficiency, while particles smaller than 5 nm are readily cleared by the blood system. NCs greater than 200 nm have shown decreased accumulation in lymphatic nodes.^141^ Therefore, nanovaccines delivery systems being developed tend to stay within the optimal cellular uptake range.

Additionally, nanovaccines can exploit the pressure difference between blood vessels and lymphatic vessels, promoting the efficient drainage of NCs into the lymphatic system.^85^ This natural drainage mechanism directs NCs toward lymphatic nodes, where they can effectively interact with APCs such as DCs. Specifically, tumor-draining lymphatic nodes (TDLNs), which are lymph nodes that filter fluid from tumor sites, have shown significant potential for nanovaccines delivery in cancer immunotherapy.^142^ As tumor antigens naturally reach TDLNs via lymphatic drainage or transport by APCs, targeting these nodes with immunostimulatory signals can potentially stimulate specific immune responses.^143^ This targeted delivery not only improves antigen presentation and T cell activation but also helps overcome the immunosuppressive environment often present in TDLNs.

### 5.2 Targeted Delivery of Antigens and Adjuvants

Precise targeting is a hallmark feature of nanotechnology.^144^ In the context of nanovaccines for cancer, the precise engineering of NCs allows for surface modifications that allows targeting specific lymphatic node receptors, enhancing uptake by DCs and other APCs.^19^ Surface functionalization involves modifying the outer layer of the NCs with specific molecules such as ligands, antibodies, or peptides that have a high affinity for receptors expressed on the surface of DCs and other immune cells, enhancing their interaction with the lymphatic environment.^145^

One common surface modification strategy involves decorating NCs with mannose or other carbohydrates that bind to lectin receptors on DCs.^88,146,147^ These receptors are naturally involved in pathogen recognition and uptake, so exploiting this pathway ensures that the NCs are efficiently captured by DCs. Another strategy is to use antibodies or antibody fragments that specifically target surface molecules on DCs, such as CD40, DEC-205 or CD11c,^91^ ensuring that the encapsulated antigens are delivered directly to the cells responsible for the immune response.

Nanovaccines can also include agents that modulate the TME itself, creating conditions that favor an immune response against cancer. For example, the inclusion of tumor necrosis factor-α (TNF-α) within the nanovaccine formulation promotes disruption of the endothelial barrier and create a significant increase in vascular permeability, increasing the likelihood of tumor cell destruction.^148^ Another example are nanovaccines that can be designed to deliver inhibitors of vascular endothelial growth factor receptors (VEGFRs).^149^ By inhibiting VEGFRs, these nanovaccines can disrupt the formation of new blood vessels that tumors need to grow and spread, effectively starving the tumor of nutrients and oxygen. With the delivery of these agents directly to the tumor site and its surrounding tissue, nanovaccines can reprogram the microenvironment to support a more effective immune response. This localized delivery minimizes systemic side effects and maximizes the therapeutic impact.

The precision targeting capability of nanotechnology not only enhances the delivery of antigens to the appropriate immune cells but also improves the overall safety and efficacy of the vaccine. By reducing the likelihood of nonspecific interactions and off-target effects, nanovaccines can elicit a more focused and potent immune response against cancer cells.^150^ This precise targeting is particularly crucial in the context of cancer immunotherapy, where the goal is to activate the immune system specifically against tumor cells while sparing healthy tissues, reducing side effects and improving patient outcomes.

### 5.3 Codelivery of Active Molecules

Another strategy employed by nanovaccines to enhance their therapeutic efficacy in cancer treatment is the codelivery of active agents. By simultaneously delivering multiple therapeutic agents, nanovaccines can address various aspects of tumor biology and immune response, creating a more comprehensive and potent anticancer effect.^151−153^ This technique relies on the unique capabilities of nanotechnology to encapsulate and protect diverse bioactive molecules, ensuring their coordinated release at the target site, enhancing treatment efficacy while minimizing the risk of resistance development.

One of the primary advantages of codelivery is the ability to combine antigens with adjuvants within a single NCs. Antigens trigger an immune response, while adjuvants enhance the strength and duration of such response. In traditional vaccines, these components are often administered separately, which can lead to suboptimal immune activation.^29^ Nanovaccines, however, can encapsulate both antigens and adjuvants together, ensuring their simultaneous delivery to DCs and other APCs.^152^

The versatility of nanovaccine platforms also allows for the inclusion of chemotherapeutic drugs, genetic material such as siRNA or mRNA, and even other NCs within a single formulation.^154−156^ Recent research has demonstrated that combining chemotherapeutics capable of inducing immunogenic cell death with immunotherapy can significantly enhance cancer immunity.^157,158^ This multimodal technique can address different aspects of cancer progression simultaneously, such as directly killing cancer cells, preventing metastasis, and enhancing immune surveillance.

### 5.4 Enhanced Adaptive Immune Response

Overcoming immunosuppression is a critical element in nanovaccine design for cancer therapy, as the TME often employs various mechanisms to evade immune surveillance and inhibit antitumor immune responses.^159^ Examples of evasion mechanisms are the secretion of immunosuppressive cytokines such as TGF-β and IL-10, the recruitment of regulatory T cells (Tregs) and myeloid-derived suppressor cells (MDSCs), and the expression of immunosuppressive molecules like PD-L1.^159−163^ These factors collectively contribute to tumor evasion of immune surveillance and uncontrollable growth.

Nanovaccines can be engineered to counteract these immunosuppressive strategies effectively. One method involves the use of nanovaccines in combination with immune checkpoint inhibitors, such as anti-PD-1/PD-L1 and anti-CTLA-4 antibodies, which block the pathways that tumors use to suppress T cell activity.^164^ By inhibiting these checkpoints, nanovaccines can recall T cells, restoring their ability to attack and kill cancer cells.^165^ In the clinic, nanovaccines and immune checkpoint inhibitors are usually administered separately, i.e., nanovaccines intramuscularly, and inhibitors intravenously, following different dosing schedules and cycles. Moreover, recent studies are exploring engineered nanovaccines that not only encode these inhibitors but also perform their additional functions.^166^ Additionally, nanovaccines can be designed to deliver agents that modulate the TME,^151^ such as small molecule inhibitors or siRNA to silence genes, while protecting the cargo from degradation and extending their half-life, further enhancing the antitumor immune response.^167^

The induction of immunological memory is another critical goal in enhanced adaptive immune response, and nanovaccines are able to enhance this process through several mechanisms^168^ A key aspect of inducing immunological memory is the formation of memory T cells. These cells can quickly express effector genes and mount an effective immune response upon re-exposure to the same antigen without the need for differentiation.^169^ Nanovaccines improve this process, where the immune system is more efficiently and directly exposed to the immunological agents over an extended period, leading to the generation of a robust memory response.^102^

Furthermore, nanovaccines can be designed to include specific signals that favor activation of T cells, including cytokines such as IL-15, which are known to promote the survival and maintenance of T cells.^151^ By providing these supportive signals, nanovaccines ensure that a substantial pool of memory T cells is generated and maintained, ready to respond to future cancer challenges. These memory T cells, along with memory B cells capable of rapidly producing antibodies, provide another layer of protection against cancer recurrence.

### 5.5 Endosomal Escape

When internalized by endocytosis, NCs are transferred to early endosomes that undergo a maturation process until they become lysosomes, known for having an acidic lumen favorable for the enzymatic degradation of foreign materials.^170^ Endosomal escape refers to the process of escaping from the endosome before it matures to prevent the degradation of the active component.^170^ This process is particularly important for mRNA vaccines since the failure to release mRNA into the cytoplasm prevents its translation into the protein of interest and, therefore, the processing for antigen presentation to CD8^+^ T and CD4^+^ T lymphocytes.^170,171^ Currently, a very limited amount of payload is released into the cytoplasm by FDA-approved LNPs.^170,171^ Various strategies have been developed to improve endosomal escape, such as pH-responsive polymeric NCs, modifications with fusogenic and cell-penetrating peptides, and the application of ionizable lipids that become protonated in acidic pH, damaging the endosome by reacting with anionic lipids present on the luminal side of its membrane.^126,129,130,138,170^ This process is not completely understood and represents a significant challenge for improving the efficacy of nanovaccines.^172^

## 6 Approved Vaccines and Clinical Trials

### 6.1 Approved Cancer Vaccines

FDA has approved five vaccines for cancer so far: TICE BCG, based on Bacillus Calmette-Guérin, a form of the bacterium *Mycobacterium bovis* that does not cause disease, used to prevent tuberculosis and treat bladder cancer;^173^ Sipuleucel-T (Provenge), an autologous cellular immunotherapy targeting prostate acid phosphatase (PAP), used to treat metastatic prostate cancer; Cevarix, a vaccine approved for preventing cervical cancer related to HPV 16 and HPV 18; Gardasil, approved for the same cancer type but targets HPV 06, 11, 16, and 18;^174,175^ and Gardasil 9, approved in 2014 to prevent infection and disease caused by nine HPV types, including seven types that cause cervical and other cancers.^176^Table 2 summarizes these approved cancer vaccines.

**Table 2:** Summary of FDA Approval of Cancer Vaccines^a^

name	vaccine type	active component	approval year	cancer type	sponsor
TICE BCG	therapeutic	attenuated strain of the bacterium *Mycobacterium bovis*	1990	bladder cancer	Merck & Co., Inc.
Provenge (Sipuleucel-T)	therapeutic	autologous PBMC	2010	metastatic prostate cancer	Dendreon Pharmaceutics
Gardasil	preventive	HPV-VLP 16/18	2006	cervical cancer	Merck & Co., Inc.
Cervarix	preventive	HPV-VLP 06/11/16/18 VLP	2009	cervical cancer	GlaxoSmithKline
Gardasil 9	preventive	HPV-VLP 06/11/16/18/31/33/45/52/58	2014	cervical, anal, penile, vaginal, vulvar, and oropharyngeal cancers	Merck & Co., Inc.

a Abbreviations: PBMC, peripheral blood mononuclear cell; VLP, virus-like particle.

### 6.2 Clinical Trials

#### 6.2.1 Phase I

During Phase 1 of clinical trials with nanovaccines, researchers assess the safety and dosage levels of the experimental treatment in a small group of participants. These trials are crucial for identifying any adverse side effects and determining the maximum tolerated dose. The primary goal is to establish a safe foundation for the subsequent phases of the trial, where the efficacy and extent of clinical benefit will be explored in larger populations.

An ongoing Phase I clinical trial (NCT05714748) is evaluating the efficacy and safety of a LNPs encapsulated mRNA vaccine for Epstein–Barr virus (EBV)-positive advanced malignancies. The project includes a Phase I clinical trial aimed at developing a candidate therapeutic vaccine with independent intellectual property.

Another Phase 1 clinical trial (NCT05475106) is currently underway to evaluate the effectiveness of personalized neoantigen peptide vaccines combined with Leukine (Sargramostim) in cancer patients. The study involves intradermal injections of personalized multipeptide vaccines with Leukine over weeks, aiming to assess safety and efficacy in patients with neoantigens. Approved by the Ethics Committee, the trial adheres to the Declaration of Helsinki.

RNA lipid particle (RNA-LP) vaccines are also being evaluated in Phase I clinical trials for adult patients newly diagnosed with glioblastoma (GBM) (NCT04573140). Participants undergo surgery and chemoradiation as standard treatment for GBM, with tumor material collected for RNA extraction and loading into liposomes. RNA-LP vaccination begins 4 weeks after radiation, with patients receiving three vaccines every 2 weeks, followed by 12 monthly doses. Treatment can continue for up to 14 months, and participants are monitored until death, with magnetic resonance imaging and clinical assessments performed every 3 months for the first-year postimmunotherapy, and every 6–12 months thereafter for up to 2 years.

#### 6.2.2 Phase II

In Phase II clinical trials, the studies focus on determining the efficacy of nanovaccines in a larger group of patients, monitoring the duration of the immune response, and evaluating the impact of the vaccines on disease progression. Additionally, they aim to assess safety profiles and the potential synergistic effects of combination therapies, such as vaccines and chemotherapy/radiotherapy, or different targets within the same vaccine.

The Phase I/II study (NCT00952692) evaluated the safety and immunological response of the immune agent dHER2+AS15 ASCI combined with lapatinib, an FDA-approved drug, in patients with metastatic breast cancer, particularly those with human epidermal growth factor receptor (HER2)-overexpressing tumors resistant to trastuzumab. The dHER2+AS15 ASCI comprised a recombinant protein, dHER2, a truncated version of the HER2 protein, combined with the AS15 adjuvant system, containing three immunostimulatory components—monophosphoryl lipid A (MPL) and CpG ODN, which are TLR agonists, and Quillaja saponaria Molina fraction 21 (QS21), an activator of immune cells—in a liposomal formulation. Patients received dHER2+AS15 ASCI injections intramuscularly every 2 weeks in 2 cycles, with a 4-week interval between cycles. Each 500 μg dose of dHER2+AS15 ASCI was reconstituted with a liquid adjuvant diluent, while lapatinib was taken concurrently, orally, as five tablets (1250 mg) per day for 43 weeks. While dHER2+AS15 ASCI stimulates the immune system to recognize and attack HER2+ tumor cells, lapatinib, a dual tyrosine kinase inhibitor that targets HER1 and HER2, blocks the growth signaling of these cells.^177^

Meanwhile, the Phase II study (NCT00828009) investigates the safety of administering the BLP25 liposomal vaccine (tecemotide) alongside bevacizumab following chemotherapy and radiotherapy in patients with stage IIIA or IIIB nonsmall cell lung cancer (NSCLC). The rationale behind this study lies in the potential synergy between the vaccine therapy, which helps the body to develop an immune response to fight tumor cells, and bevacizumab, a monoclonal antibody known to prevent tumor growth through several mechanisms. The study aims to determine the safety profile of such combination treatment regimen and evaluate key outcomes such as overall survival and progression-free survival.

The ongoing Phase II clinical trial (NCT04580771) aims assess the safety and toxicity profile of administering the immune nanoparticle liposomal HPV-16 E6/E7 multipeptide vaccine PDS0101 with standard-of-care chemoradiation in patients with locally advanced cervical cancer. The PDS0101 vaccine is designed to enhance the immune response to HPV16-infected cervical tumor cells, and its combination with chemoradiation may improve the management of cervical cancer.

#### 6.2.3 Phase III

In Phase III trials, clinical studies are designed to confirm the effectiveness of a new treatment and further evaluate its safety, involving a larger group of participants. These studies compare the new treatment against the current standard therapy, aiming to provide definitive evidence of its efficacy and monitor side effects in a diverse population.

In the ongoing Phase II/III study NCT05141721, the Phase II portion will assess the clinical activity of maintenance therapy using two vectors in a heterologous prime/boost approach (GRT-C901 followed by GRT-R902) to stimulate a robust immune response in combination with checkpoint inhibitors, along with fluoropyrimidine/bevacizumab, compared to fluoropyrimidine/bevacizumab alone in patients with advanced cancer. Meanwhile, the Phase III portion aims to demonstrate the clinical efficacy of the combined therapy regimen, using progression-free survival as the primary metric, and also to evaluate key clinical outcomes, such as overall survival and progression-free survival.

Two Phase III studies are currently recruiting participants to evaluate the efficacy of V940, an individualized neoantigen therapy, in combination with pembrolizumab for different cancer indications. The first study (NCT05933577) investigates V940, previously known as mRNA-4157, combined with pembrolizumab in preventing cancer recurrence in patients with high-risk melanoma. This study aims to determine if this combination is more effective than pembrolizumab alone in preventing cancer from returning. The second study (NCT06077760) assesses the efficacy of V940 combined with pembrolizumab versus a placebo with pembrolizumab in the adjuvant treatment of completely resected Stage II, IIIA, and IIIB (N2) NSCLC. This randomized, double-blind trial aims to determine if V940 enhances disease-free survival compared to the control group receiving placebo and pembrolizumab. Participants will receive 1 mg of V940 intramuscularly every 3 weeks (nine doses), along with 400 mg of pembrolizumab intravenously every 6 weeks (nine doses). The study is designed to continue until disease recurrence, the emergence of unacceptable toxicity, or for a maximum treatment duration of approximately one year. Table 3 summarizes the cancer nanovaccines in Phase I, II, and III and clinical phase.

**Table 3:** Summary of Cancer Nanovaccines in the Clinical Phase^a^

clinical trial stage	proprietary name	delivery system	active molecules	combination therapy	cancer type	sponsor	NCT number	status
Phase 1	PDS0101	liposomal	HPV-16 E6 and E7 peptides		cervical intraepithelial neoplasia and high-risk HPV infection	PDS Biotechnology Corp.	NCT02065973	completed
	DPX-Survivac		surviving peptide	low-dose cyclophosphamide and DPX-Survivac (aqueous)	surgically operable or advanced stage ovarian, fallopian tube or peritoneal cancer	ImmunoVaccine Technologies, Inc.	NCT03332576	active, not recruiting
				low-dose cyclophosphamide			NCT01416038	completed
				cyclophosphamide and Epacadostat	recurrent ovarian cancer		NCT02785250	active, not recruiting
	DPX-0907		7 tumor-specific HLA-A2-restricted peptides, a universal T helper peptide and a polynucleotide adjuvant		ovarian, breast, and prostate cancer in advanced stage		NCT01095848	completed
	DPX-E7		E7 peptide of the HPV-16 protein	cyclophosphamide	HPV-16- related oropharyngeal, cervical, and anal cancer	Dana-Farber	NCT02865135	active, not recruiting
						Cancer Institute		
	dHER2+AS15		truncated HER2 protein combined with the immunological liposomal AS15 adjuvant		HER2-positive metastatic breast cancer	GlaxoSmithKline (GSK)	NCT00140738	completed
	Lipovaxin-MM	DC-targeted liposome	melanoma antigens		malignant melanoma	Lipotek Pty Ltd.	NCT01052142	completed
	WGc-043	LNPs	EBV mRNA		EBV-positive advanced malignant tumor	West China Hospital	NCT05714748	recruiting
	RNA-LP	liposome (DOTAP)	autologous total tumor mRNA and pp65 full length (fl) lysosomal-associated membrane protein (LAMP) mRNA		newly diagnosed pediatric high-grade gliomas and adult glioblastoma	University of Florida	NCT04573140	recruiting
	IVAC_W_bre1_uID e IVAC_M_uID	liposomal	mRNA encoding TAAs (IVAC_W_bre1_uID) and mRNAs targeting up to 20 individual tumor mutations (IVAC_M_uID)		triple negative breast cancer	BioNTech SE	NCT02316457	completed
	BNT116	Lipoplex (liposomal)	six mRNAs encoding MAGE A3, CLDN6, KK-LC-1, PRAME, MAGE A4, and MAGE C1	Cemiplimab and Docetaxel	advanced non-small cell lung cancer		NCT05142189	recruiting
Phase I/2	PDS0101	liposomal	HPV-16 E6/E7Multipeptide	Pembrolizumab	human papillomavirus-associated oropharynx cancer	Mayo Clinic	NCT05232851	recruiting
	mRNA-4359	LNP	mRNA-derived IDO/PD-L1-targeted	Pembrolizumab	advanced solid tumors	ModernaTX, Inc.	NCT05533697	recruiting
Phase 2	PDS0101	liposome (DOTAP)	HPV-16 E6/E7Multipeptide	cisplatin and radiation therapy	Stage IB3-IVA cervical cancer	M.D. Anderson Cancer Center	NCT04580771	active, not recruiting
	Tecemotide (L-BLP25)	liposomal	MUC1 antigen	Bevacizumab and Carboplatin	unresectable Stage IIIA and IIIB non-squamous non-small cell lung cancer	Merck KGaA	NCT00828009	active, not recruiting
					colorectal carcinoma after curative resection of hepatic metastases		NCT01462513	completed
				cyclophosphamide and chemoradiotherapy	rectal cancer		NCT01507103	completed
				radiation therapy, goserelin, and cyclophosphamide	untreated, intermediate and high risk prostate cancer		NCT01496131	completed
				cyclophosphamide	slowly progressive multiple myeloma with no symptoms and who have had no chemotherapy		NCT01094548	completed
	DPX-Survivac	liposomal	surviving peptide	Pembrolizumab and cyclophosphamide	advanced ovarian, primary peritoneal or fallopian tube cancer	ImmunoVaccine Technologies, Inc.	NCT03029403	recruiting
				cyclophosphamide	recurrent survivin-expressing diffuse large B-cell lymphoma (DLBCL)		NCT02323230	active, not recruiting
				Pembrolizumab and cyclophosphamide	persistent or recurrent/refractory diffuse large B-cell lymphoma		NCT03349450	recruiting
	dHER2+AS15	liposomal	AS15 (immunological liposomal adjuvant) and truncated HER2 protein	Lapatinib	HER2-positive metastatic breast cancer	GlaxoSmithKline (GSK)	NCT00952692	completed
	V940-005 (INTerpath-005)	LNP	single synthetic mRNA coding for up to 34 neoantigens	Pembrolizumab	high-risk muscle-invasive urothelial carcinoma postradical resection	Merck Sharp & Dohme LLC	NCT06305767	recruiting
	V940-004 (INTerpath-004)				renal cell carcinoma		NCT06307431	recruiting
	BNT111	Lipoplex (liposomal)	mRNA encoding melanoma-associated antigens (NY-ESO-1, MAGE-A3, tyrosinase, and TPTE)	Cemiplimab	unresectable Stage III or IV melanoma	BioNTech SE	NCT04526899	active, not recruiting
	BNT113		mRNA encoding HPV-16 oncoproteins E6 and E7	Pembrolizumab	unresectable recurrent, or metastatic head and neck squamous cell carcinoma and positive for HPV16		NCT04534205	recruiting
	BNT116		six mRNAs encoding MAGE A3, CLDN6, KK-LC-1, PRAME, MAGE A4, and MAGE C1	Cemiplimab	advanced non-small cell lung cancer with tumors expressing PD-L1 ≥ 50%	Regeneron Pharmaceuticals	NCT05557591	recruiting
	BNT122 (autogene cevumeran)		mRNA encoding for up to 20 MHC class I and class II restricted neoantigens		resected, Stage II (high risk) and Stage III colorectal cancer ctDNA positive	BioNTech SE	NCT04486378	recruiting
				Pembrolizumab	previously untreated advanced melanoma	Genentech, lnc.	NCT03815058	completed
				Atezolizumab	locally advanced or metastatic tumors		NCT03289962	active, not recruiting
				Atezolizumab and mFOLFIRINOX	resected pancreatic ductal adenocarcinoma		NCT05968326	recruiting
Phase 2/3	V940-007 (INTerpath-007)	LNP	single synthetic mRNA coding for up to 34 neoantigens	Pembrolizumab	resectable locally advanced cutaneous squamous cell carcinoma	Merck Sharp & Dohme LLC	NCT06295809	recruiting
	GRANITE-001	adenoviral vector/LNP	GRT-C901 (adenoviral tumor-specific neoantigen priming vaccine) and GRT-R902 (self-amplifying mRNA formulated in a LNP)	Atezolizumab	metastatic colorectal cancer	Gritstone bio, Inc.	NCT05141721	active, not recruiting
Phase 3	V940-001 (INTerpath-001)	LNP	single synthetic mRNA coding for up to 34 neoantigens	Pembrolizumab	high-risk Stage II–IV melanoma	Merck Sharp & Dohme LLC	NCT05933577	recruiting
	V940-002 (INTerpath-002)				resected Stage II, IIIA, IIIB (N2) non-small cell lung cancer		NCT06077760	recruiting

a Abbreviations: CEA, carcinoembryonic; DC, dendritic cell; HER2, human epidermal growth factor receptor 2; HLA, human leukocyte antigen; HPV, human papillomavirus; MHC, major histocompatibility complex; MPLA, monophosphoryl lipid A; MUC1, Mucin 1; TAA, tumor-associated antigen.

### 6.3 Challenges in Clinical Translation

Cancer nanovaccines face similar challenges to other nanomedicines in their translation to the clinic, including biological, regulatory, and technological barriers that significantly impact their biodistribution, efficacy, large-scale production, and distribution. A major biological challenge lies in the interaction of nanoparticles with biological components, leading to the formation of a biomolecular corona composed mainly of proteins, lipids and carbohydrates.^178^ These biomolecules adsorbed on the nanoparticle’s surface influence biodistribution, cellular uptake, and therapeutic efficacy.^179^ Therefore, standardized characterization methods are needed to gain a deeper understanding of their implications,^178^ considering the impact of variability among patients with different health profiles. In the preclinical context, the need for more representative experimental models is critical to reducing the discrepancy between *in vitro*, *in vivo*, and clinical data. Models such as *organs-on-a-chip*, *human-on-a-chip*, and three-dimensional spheroids stand out for their ability to mimic living systems more accurately.^178,180^ Additionally, artificial intelligence-based *in silico* models integrating *in vitro* and *in vivo* data can enable more robust simulations and accelerate formulation optimization.^178^

Clinical translation also requires advances in scaling up and manufacturing in compliance with good manufacturing practices (GMPs). Batch-to-batch reproducibility and standardization of nanoparticle quality are critical challenges that can be addressed by implementing autonomous manufacturing processes and artificial intelligence to optimize synthesis parameters, monitor quality in real time, and reduce variability in production.^178,181^ From a regulatory perspective, the challenge lies in achieving international harmonization of regulatory standards and evaluation methods for nanomedicines, ensuring greater guidance and clarity in the approval process.^178^ These efforts are crucial to preventing the full potential of nanomedicine from being hindered by regulatory framework issues.^182^ At the same time, efforts are being made to simplify, scale up, and automate processes to overcome the high costs of nanomedicines compared to conventional drugs, despite their justified cost-effectiveness.^178,183^ Achieving more affordable prices and expanding access for end consumers and hospitals could significantly impact healthcare expenditures by allowing the replacement of ineffective treatments that impose substantial costs.

**Figure 3:**
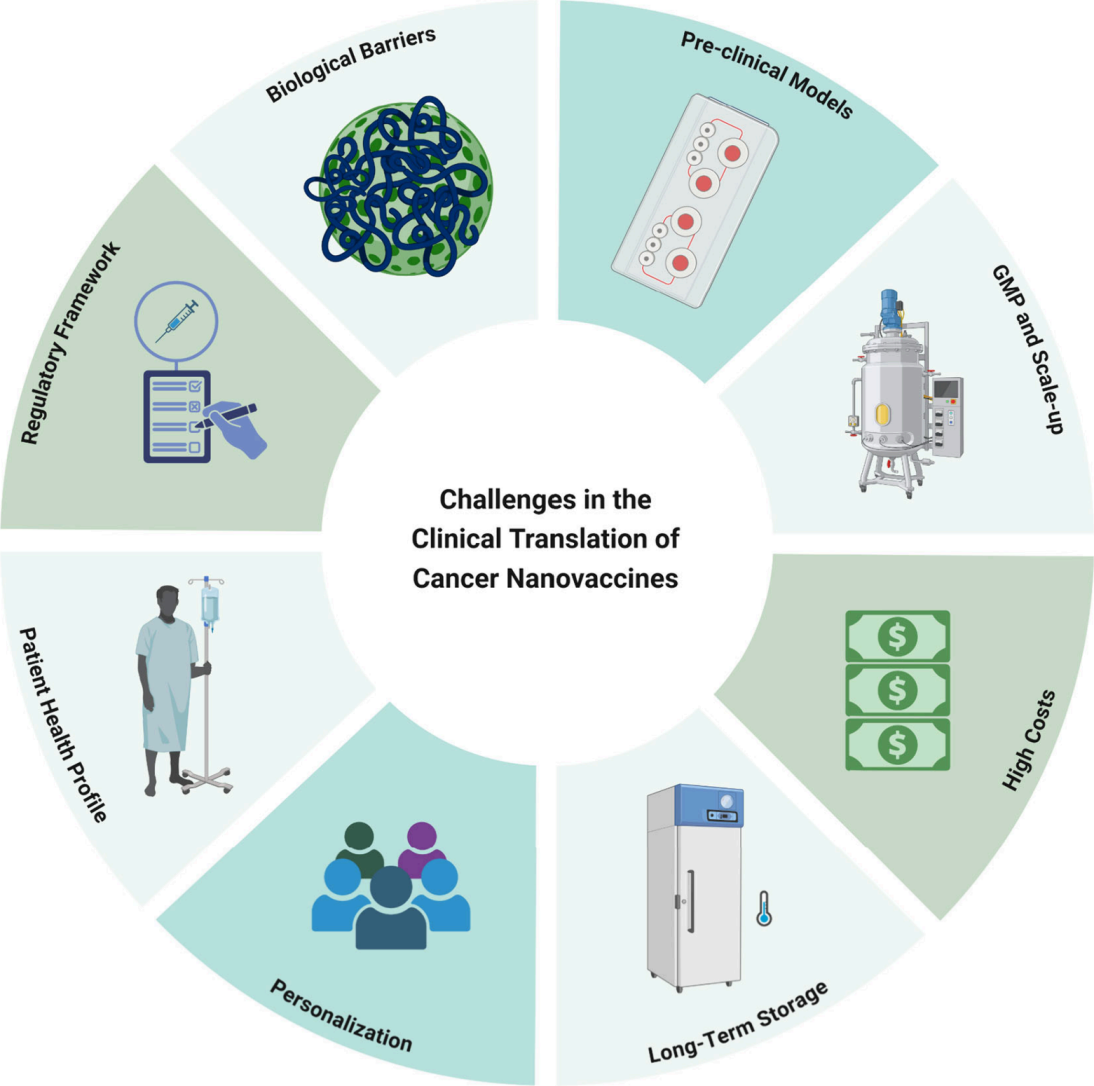
Main challenges regarding the clinical translation of cancer nanovaccines. Created by BioRender.com.

Cancer nanovaccines also face specific obstacles. The variability in immune responses among patients is a critical factor, especially considering that individuals who have undergone prior treatments, such as chemotherapy, may have impaired adaptive immune system functionality,^184^ affecting vaccination efficacy. For *off-the-shelf* nanovaccines, the long-term formulation stability represents a significant challenge. A deeper understanding of optimal storage conditions and interactions among formulation components is essential to ensure therapeutic activity and safety.^185,186^ For personalized nanovaccines, challenges include establishing standardized customization protocols and reducing production time—key factors for clinical implementation. Moreover, efficient selection of immunogenic antigens requires the development of more precise machine learning models integrated with experimental data to reliably predict neoantigens for application.^187^Figure 3 depicts a scheme of the main challenges regarding the clinical translation of cancer nanovaccines.

## 7 Conclusion and Perspectives

Cancer nanovaccines represent a promising frontier in immunotherapy, offering innovative strategies to overcome traditional challenges associated with oncological treatment. This review highlighted recent advances in the formulation and application of nanovaccines, demonstrating their ability to induce specific immune responses against tumor antigens and their potential effectiveness in enhancing the immune system’s ability to combat tumors. Despite the recent advancements, significant challenges remain, such as optimizing targeted delivery, overcoming the immunosuppression of the TME, and the need for robust clinical trials to validate the efficacy and safety of these therapies. Future actions include the integration of emerging technologies, such as artificial intelligence, multiomics, and synthetic biology, to develop more effective nanovaccines. Additionally, combining nanovaccines with other therapeutic strategies, such as checkpoint inhibitors and CAR-T therapy, promises to generate novel methods that can significantly enhance the antitumor response. However, it is worth emphasizing that the development of new strategies should always be guided by the pursuit of simplicity, facilitating scalability and reducing the costs of the final product. In addition, sharing data in open repositories for the standardization of characterization protocols, optimization of formulations, and validation of efficacy can drive progress in the field and enable its application in AI models. Furthermore, while it is essential to refine already established NCs, such as lipid-based nanoparticles, the search for innovations in nanomaterials remains highly relevant. In clinical settings, implementing patient stratification strategies for targeted recruitment can improve clinical trial outcomes and open new research opportunities for individuals with low responsiveness to nanovaccine. Therefore, as research progresses to make nanovaccines a viable option for cancer treatment, interdisciplinary collaboration among researchers, clinicians, industry, and regulatory agencies will be crucial to accelerate the translation of these innovations into clinical practice.

## Vocabulary

cancer nanovaccines: nanocarrier-based vaccination platforms for the targeted delivery of tumor antigens and adjuvants, aiming to activate the adaptive immune system against tumor cells
nanocarriers: nanoscale delivery systems composed of synthetic, semisynthetic, or biological nanomaterials, designed for the targeted delivery and sustained release of active agents of interest
molecular adjuvants: immunomodulatory molecules capable of enhancing antigen immunogenicity by stimulating adaptive and innate immune system pathways
lymphatic system: key component of the body’s immune system, consisting of a network of tissues and organs such as lymph nodes, lymph vessels, bone marrow, spleen, among others, where antigen presentation and immune cell activation occur; it helps to fight infections and diseases while maintaining fluid balance and removing cellular debris and harmful substances from tissues
adaptive immune system: part of the immune system that generates an antigen-specific response, playing a crucial role in responding to foreign substances, microorganisms, and tumor cells; it involves specialized immune cells, including APCs, T and B cells, and antibodies, that recognize and eliminate threats while creating immune memory
